# Consumers and Carer perspectives on poor practice and the use of seclusion and restraint in mental health settings: results from Australian focus groups

**DOI:** 10.1186/s13033-016-0038-x

**Published:** 2016-02-06

**Authors:** Lisa M. Brophy, Catherine E. Roper, Bridget E. Hamilton, Juan José Tellez, Bernadette M. McSherry

**Affiliations:** Centre for Mental Health, Melbourne School of Population and Global Health, University of Melbourne, 4/207 Bouverie Street, Carlton, VIC 3010 Australia; Mind Australia, 86-92 Mount Street, Heidelberg, VIC 3084 Australia; Consumer Academic, Centre for Psychiatric Nursing, School of Health Sciences, University of Melbourne, Level 6 Alan Gilbert Building, 161 Barry Street, Carlton, VIC 3053 Australia; Department of Nursing, School of Health Sciences, University of Melbourne, Level 6 Alan Gilbert Building, 161 Barry Street, Carlton, VIC 3053 Australia; St Vincent’s Mental Health, 41 Victoria Parade, Fitzroy, VIC 3065 Australia; Melbourne Social Equity Institute, University of Melbourne, 201 Grattan Street, Carlton, VIC 3053 Australia; Melbourne Law School, University Square, 185 Pelham Street, Carlton, VIC 3035 Australia; Faculty of Law, Monash University, 15 Ancora Imparo Way, Wellington Road, Clayton, VIC 3800 Australia

## Abstract

**Background:**

Seclusion and restraint are interventions currently permitted for use in mental health services to control or manage a person’s behaviour. In Australia, serious concerns about the use of such seclusion and restraint have been raised at least since 1993. Consumers and their supporters have also expressed strong views about the harm of these practices. This paper presents the results of ten focus group discussions with people with lived experience of mental health issues and also carers, family members and support persons in relation to the use of seclusion and restraint.

**Methods:**

The 30 consumers and 36 supporters participating in the focus groups convened in four Australian cities and one regional centre discussed their understandings of the use of seclusion and restraint and its impact on the people involved. Participants also presented their observations about poor practice and what contributes to it as well as providing ideas and recommendations regarding strategies to reduce or eliminate seclusion and restraint. Focus group discussions were recorded and transcribed, then analysed using the NVivo 10 qualitative data analysis software with a general inductive approach used to analyse data. This analysis enabled consideration of the responses to key questions in the focus groups as well as the identification of emerging themes.

**Results:**

Six themes emerged from the analysis, these being: human rights, trauma, control, isolation, dehumanisation and ‘othering’, and anti-recovery. Examples of poor practice identified by focus groups included the use of excessive force, lack of empathy/paternalistic attitudes, lack of communication and interaction and a lack of alternative strategies to the use of seclusion and restraint. There was a confluence of factors identified by participants as contributing to poor practice, with the main factors being organisational culture, the physical environment, under-resourced mental health services and fear and stigma.

**Conclusions:**

Focus group participants in the main viewed seclusion and restraint practices in mental health settings as unnecessarily overused, exacerbating problems for individuals, carers, staff and the broader system of care. This study highlights that lived experience of both consumers and their supporters can make an important contribution to mental health services and its ongoing reform.

## Background

In Australia, the six states and two territory governments fund and deliver public sector mental health services that provide specialist care for people with severe mental health problems. It is estimated that 2–3 % of Australians (about 600,000 people) have a diagnosis of a severe mental disorder defined as including severe depression, anxiety or psychosis [1] and that approximately 0.5 % have some form of psychotic disorder, most often diagnosed as schizophrenia [2]. Each state and territory has mental health legislation that enables the compulsory detention and treatment for those with severe mental health problems providing strict criteria are met [3].

Seclusion and restraint are interventions currently permitted for use in mental health services and other settings to control or manage a person’s behaviour. Seclusion generally refers to the deliberate confinement of a person, alone, in a room or area that he or she cannot freely exit. Each state and territory regulates the use of seclusion through its mental health legislation [4]. Rates of seclusion are falling in Australia with 7.8 seclusion events estimated per 1000 bed days in 2014–2015; a decrease from 11.8 in 2010–2011 [5].

Restraint may encompass the use of bodily force (physical restraint) or a device (mechanical restraint) to control a person’s freedom of movement. It may also refer to the use of medication (chemical restraint) to control a person’s behaviour rather than to treat a mental disorder. Despite the possible adverse effects of physical and mechanical restraint, only mechanical restraint is regulated in every state and territory under mental health legislation [6]. Physical restraint is regulated under policies and/or mental health legislation in the six states and the Australian Capital Territory, but is not regulated at all in the Northern Territory. The rates of restraint are difficult to estimate in Australia due to differences in reporting requirements across the states and territories and the current lack of national data collection.

Serious concerns about the use of seclusion and mechanical restraint in mental health services have been raised at least since 1993 [7]. There have been adverse findings by investigators regarding serious injuries resulting from the use of bodily force [8] as well as concerns raised about deprivations of liberty, interference with personal integrity and loss of dignity. A number of studies have noted adverse consequences for those subjected to seclusion and mechanical restraint [9, 10] and raised concerns with human rights breaches [4].

In 2008, the National Mental Health Policy set out that Australian mental health services should adopt a recovery-oriented approach [11] and there is now a National Framework for Recovery-Oriented Mental Health Services [12]. In tandem with the recovery movement, an emphasis on human rights is shaping mental health law reform [13, 14]. The Convention on the Rights of Persons with Disabilities, which Australia has ratified sets out as its first guiding principle in Article 3, ‘[r]espect for inherent dignity, individual autonomy including the freedom to make one’s own choices’. This emphasis on recovery and rights provides an ethical conceptual framework for viewing the use of seclusion and restraint as inherently problematic [15]. As outlined below, the harmful effects of seclusion and restraint raise issues as to whether recovery-oriented practice and the use of seclusion and restraint are compatible [16].

Consumers of mental health services (‘consumers’) have expressed strong views about the harm of seclusion and all forms of restraint [17]. Consumers and their families, friends and other supporters (‘supporters’) have also raised concerns with what has been termed ‘emotional restraint’ whereby consumers feel constrained from expressing their views openly and honestly to staff for fear of the consequences [18]. Emotional restraint in this sense is about been pressured to comply with behavioural expectations.

Judi Chamberlin has made the point that for those ‘labelled with mental illness…outright prejudice and discrimination…is just as real, and as disabling, as those faced by other devalued groups’ [19]. While she acknowledges ‘stigma’ is often used in relation to the experiences of consumers, she points out that this word locates the problem as within the individual [19]. In order to counteract emotional restraint, prejudice and discrimination, it is essential that the perspectives of consumers and their supporters are aired and valued in a system which may be based as much on coercion as care.

This paper reports on the outcomes of ten focus groups in which consumers and their supporters discussed the effects of seclusion and restraint and what consumers and supporters viewed as ‘poor practice’ and its causes leading to the use of these interventions. ‘Poor practice’ is used in this paper as a general term to describe behaviour identified as unacceptable by consumers and their supporters.

Since the late 1990s a number of qualitative interview studies, spread across a range of individual inpatient settings, have been conducted in relation to consumer perspectives on seclusion and mechanical restraint internationally [20–23]. While these have conveyed vivid accounts of consumer experience, the lack of detail about the legal or practice context for the use of seclusion and restraint can limit the opportunity for comparison across jurisdictions. Several other studies have used questionnaires [24–27] and in these studies the samples are also specific to one service, with the exception being one study which compared psychiatric services across New York state [27]. Studies vary widely in how soon after seclusion or restraint events people are interviewed, ranging from 3 days after to many years later. Despite their diversity, these studies raise many shared concerns.

As noted in a recent review [28], consumer experiences of physical restraint and seclusion are reported as overwhelmingly negative, associated with immediate escalation of distress, and intense feelings such as despair, shame, terror and rage [29]. Patients reported feeling: frightened, anxious, angry, helpless, humiliated and vulnerable, re-traumatised by the experience [23]; abandoned, deserted, excluded and rejected [20]. Harms were experienced before, during and after the incidents [30]. Other detailed impacts include: a sense of injustice [21], and of being punished and powerless [22]. A significant subset of participants reported experiences strongly suggestive of poor practice in the use of seclusion and restraint, such as patronising communication, physical harassment and insulting communication [32] and extreme use of force [22]. In one US study, participants reported a sense of being both punished and abandoned by staff, particularly in relation to ‘non-professional’ (security/attendant) staff [33].

A small number of studies, or a minority of consumer participants within studies, report positive views, such as: that seclusion provided an opportunity for meditation [33]; or that the use of restraint had a calming effect [23]. In one Canadian study, patients reported on comfort and safety of seclusion rooms and the meeting of their physical needs [26].

The negative effects experienced overall can obscure differences in experiences, between people and settings. In this regard, it may be that there are important differences between poor and better practices, and the associated impacts may differ.

There is a dearth of literature dealing with the perspectives of supporters on the topic of seclusion and restraint. Recent studies explore ‘carer’ views about coercion in community settings [16, 34]. These studies raise concerns about the nature and experience of social control and the lack of human responsiveness to emerging crises that are later managed via acts of coercion.

In summary, there is a gap in the literature pertaining to a broad range of consumers’ and their supporters’ perspectives on the use of seclusion and restraint. Most of the studies concerning consumers outlined above have been conducted within a single jurisdiction and mainly from a single site. There is also a lack of studies concerning supporters’ perspectives. As indicated above, despite the growing emphasis on recovery and rights, this gap may be the result of a system that reflects societal and organisational power dynamics [15].

To fill this gap, as part of a larger study conducted by the Melbourne Social Equity Institute at the University of Melbourne and funded by Australia’s National Mental Health Commission, five focus groups for consumers and five focus groups for supporters were conducted in Melbourne, Shepparton, Perth, Brisbane and Sydney. This paper focuses on the discussions concerning the effects of seclusion and restraint on consumers and supporters and what the participants thought was poor practice in this regard and what contributed to it.

## Methods

The project passed through a rigorous ethics approval process at the University of Melbourne (Ethics ID 1340647), being considered first by the Population and Global Health Human Ethics Advisory Group and then by the Health Sciences Human Ethics Sub-Committee. The project methodology was guided by two Advisory Groups, one consisting of five people with lived experience of seclusion and restraint and the other consisting of five supporters of those with lived experience of these interventions.

### Sample and data collection

Focus groups were included as part of the research design for the larger study because they are a particularly good method for generating discussion and stimulating ideas. The focus groups also enabled the voices of consumers and supporters to be highlighted in the research. The ten focus groups provided a convenience rather than a representative sample, although some attempt at purposive sampling was made by holding the focus groups in four Australian state capitals and a rural location.

The supporter focus groups consisted of 36 participants (29 women and seven men) who had experienced a family member or person close to them being secluded or restrained. These included parents, siblings, marital partners and two people who had advocacy roles. The consumer focus group consisted of 30 adults (13 men and 17 women) all of whom had either experienced seclusion or restraint directly, witnessed these practices as inpatients or were consumer advocates who directly supported people who had experienced seclusion and restraint.

The focus groups were all conducted in English but participants indicated a variety of ethnic and cultural backgrounds including Vietnamese, Italian, Greek, Dutch and other European backgrounds. The participants ranged in age from 20 years old to one participant who was over 70. No Indigenous Australians attended but people involved in supporting Indigenous people did attend. Participants self-selected and opted into the groups after receiving information through peak bodies and support services (including Indigenous health organisations) in each state where the focus groups were held. The facilitators had a brief discussion with each potential participant to confirm their eligibility to take part prior to the focus groups. Participants received a $25 shopping voucher to express appreciation for attending.

### The focus groups

The focus groups were conducted by an experienced qualitative researcher and mental health practitioner (Lisa Brophy) and a consumer academic (Cath Roper). The involvement of a researcher with lived experience was a deliberate strategy to enable and support open and safe discussion of this sensitive topic. While participants were reassured that they were not being asked to speak of their personal experiences many chose to share their direct experiences and a facilitator who shared personal experience was considered to contribute to participants’ experiences being validated.

As part of the larger study, one of the main aims of the focus groups was to give people an opportunity to share their perspectives on how seclusion and restraint could be reduced or eliminated. Participants discussed their understanding of the use of seclusion and restraint and its impact on the people involved. They also presented their observations about poor practice and what contributes to it as well as providing ideas and recommendations regarding strategies to reduce or eliminate seclusion and restraint. The findings in relation to strategies to reduce or eliminate seclusion and restraint are the subject of another paper by members of the research group [35].

### Data analysis

The focus group recordings were transcribed, then analysed using the NVivo 10 qualitative data analysis software. A general inductive approach was used to analyse the data [36]. Each transcript was closely read and re-read multiple times in order to identify categories, which were coded for words, phrases and meanings in the text by one member of the research team (Juan José Tellez) who was independent of both focus group facilitators. Categories were continually refined through the analysis with coding consistency checks performed by another team member to ensure trustworthiness of the data (Lisa Brophy). Further confirmation of themes took place through team discussions that involved both facilitators. The emerging themes were also discussed with the full research team. The project’s two Lived Experience Advisory Groups for consumers and supporters also discussed and commented on the preliminary findings. The analysis enabled consideration of the responses to the key questions in the focus groups as well as identification of emerging themes

## Results

### The experience and impact of seclusion and restraint

In nine of the ten focus groups with both consumers and supporters, there was discussion about the experience and impact of seclusion and restraint. The six themes that emerged from the analysis are: human rights, trauma, control, isolation, dehumanisation and ‘Othering’, and anti-recovery. These themes are dealt with in turn.

#### Human rights

In nine groups there was considerable discussion about the impact of seclusion and restraint on consumers and their supporters. In the main, participants identified these interventions as a breach of human rights, even when it may have seemed necessary to manage risk. For example:

*‘Unfortunately, I think there is a place for it that you do need it but on the other hand it really does take away people’s rights and it’s a pretty harsh thing to do to somebody. It’s kind of a bit of a necessary evil I suppose.’ (Supporter)*

Many participants were concerned that there was a lack of accountability for human rights breaches that may have occurred in the context of seclusion and restraint. This linked to participants being aware that many consumers seemed powerless in the situation and also, because they had mental health issues, may not be believed when they complained of abuse.

*‘We’ve had people who have come in and said this happened and I don’t know why. I don’t know why they dealt with me this way and why was I thrown on the floor and injected when all I said was please don’t give me any more of that medication it makes me really, really unwell.’ (Supporter)*

#### Trauma

Many participants expressed concerns over how seclusion and restraint resulted in trauma and also how past trauma was sometimes revisited or resonated with the experience of being coerced.

*‘And I can say that my son is so traumatised by these events, that he lives in fear of being picked up at any stage. He’s marked.’ (Supporter)*

*‘…put you in a cell that has no toilet and no air and leave you there for 10 hours and then you’ll be cured, and it’s not*…*you go in there seeking help and surviving the traumas in your life, but you end up having to cope with even more trauma. It’s pointless.’ (Consumer)*

Participants made a link between the trauma experienced as a result of seclusion and restraint, and the subsequent impact this had on the person’s recovery, sense of trust in the world and relationships with service providers.

*‘So what I’ve seen with people who’ve felt, when they’ve had even a single 24 hour experience of seclusion and restraint under the mental health system, which is the door, the police, the medication, down into the whatever, the taking of the clothes, the whole lot*—*that person’s changed forever in their feeling and their relationship to the society around them. To every other state agency they’re changed, and that allows, that’s again that learned helplessness.’ (Consumer)*

#### Control

Participants discussed how seclusion and restraint were used by staff to gain control over consumers and also to manage the environments they found themselves in. The quote below captures the discussion about how control relates to both behaviour and the maintenance of ward routines in order to contain the environment.

*‘Control for me became a sort of key feature …because I guess the feeling of the medical staff was that it was out of control…so isolation was obviously a way, the other way was sort of punishment…. The other thing that I thought was interesting, and the feedback I get and being on a unit, is that the idea of medical routine, so if people are not behaving accordingly to the routine…that they need to have their obs taken, they need to have their medication done, and that’s just routine, doesn’t matter what the individual’s state of mind is, so then they have to be kind of contained within that routine.’ (Supporter)*

There were concerns about people from different culturally and linguistically diverse (CALD) communities and the impact of the use of control and its link to further stigmatising mental health issues.

*‘I’m from a CALD background and believe me the CALD community is suffering 100 fold, because often they don’t understand what’s happening to them, certainly the families aren’t involved’. (Consumer)*

#### Isolation

Many participants commented on their concerns about people being isolated and its impact on their mental health and relationship with the service.

*‘People only went near them I think to put the food tray out there with the paper plates and paper cups and things like that, they weren’t even treated properly like they couldn’t be trusted with proper cutlery and plates and things, it was just awful.’ (Supporter)*

*‘Deny people their freedom, for example if it’s restraint of freedom of movement, or the freedom to ask questions, the freedom to be able to interact with other people, I mean isolation basically is almost another form of punishment, you’ve been bad, you’ve done something wrong. I mean that’s how I see somebody being isolated. And takes that confidence away, because you must be bad so you are in isolation.’ (Consumer)*

Isolation was raised by participants as presenting a particularly negative impact for Indigenous people:

*‘Could I put another perspective*…*another form of restraint and isolation is…when traditional Aboriginal people are brought down out of their country, and placed in an environment that’s totally alien to them, so on top of their mental illness issue they’re out of context, they’re out of country, they could be in the middle of an exercise yard, but they’re still restrained, they’re still totally isolated because they can’t connect.’ (Supporter)*

#### Dehumanisation and ‘othering’

Participants identified dehumanisation as one of the contributing factors to what they identified as poor practice, why seclusion and restraint continued as an everyday practice in mental health contexts and also it emerged as a theme in relation to the experience or impact of seclusion and restraint. This could also be described as ‘othering’ in that people had to cope with times in their life when people treated them as though they were ‘sub-human’.

*‘It’s a social justice issue, because powerlessness is an injustice, it’s actually dehumanising…and it’s not just the consumer that’s in the hospital, it’s (also) the (person’s) actual carers.’ (Supporter)*

*‘You literally just get de*-*humanised and it’s sort of that once you have become part of that system you do become almost, well not completely, but treated in a sub*-*human way. You can do things that you would not normally do. If you had a cancer patient in that same situation the furore would be terrible with the treatment they receive.’ (Consumer)*

#### Anti-recovery

Finally participants also discussed the impact of seclusion and restraint as being inconsistent with or undermining personal recovery. Many were aware that recovery was otherwise having a significant influence on policy and practice in mental health services.

*‘Seclusion and restraint, the very practices themselves, are sort of very anti*-*recovery…[Recovery is] all about self*-*responsibility, self*-*direction, and then seclusion and restraint is all about someone else’s control, so it doesn’t actually sit with recovery at all.’ (Consumer)*

The negative effects on mental health in the long-term of being secluded were recounted by one participant.

*‘So it’s not the best, it’s not the nicest, yeah it’s pretty horrible…you start to lose your mind.’ (Consumer)*

The challenge of trying to maintain a sense of balance during crisis and seclusion was articulated by another participant.

*‘It’s pretty hard because you can’t even use like some of your strategies you’d use at home because you’re just in these four walls.’ (Consumer)*

### Perspectives on ‘poor practice’

Many participants attended the focus groups because they were concerned about ongoing poor practice. This theme underscored discussion concerning the harm caused by seclusion and restraint such as the denial of human rights or isolation.

Examples of poor practice included the use of excessive force, lack of empathy/paternalistic attitudes, lack of communication and interaction, and a lack of alternative strategies. These examples are outlined in turn.

#### Excessive force

The use of excessive force to combat escalation and manage risk was a practice questioned by many participants. One participant recounted the use of excessive force by multiple service providers including clinical and non clinical staff and the police:

*‘The last time it started with the police tackling me and putting me in a paddy wagon but putting me on my stomach and leaving the cuffs on that was about the worst part of it…I was saying I can do no harm and I still got tackled.’ (Consumer)*

*‘Education, and everybody talks about doctors and nurses receiving education, that’s great, but the volunteers at the hospitals and the security guards really need to be educated that because somebody’s displaying agitated behaviour does not give you the right to come and restrain them physically.’ (Consumer)*

One participant observed that behaviour management training was too focused on physically restraining people which emphasised the exercise of power by staff over consumers.

*‘That undue use of power which is sometimes invoked with trying to seclude or restrain people, it comes right down to even when they start that aggressive behaviour management training.’ (Consumer)*

#### Lack of empathy

Lack of compassion and empathy were noted by participants as representing a lack of connection between staff and patients.

*‘I’ve seen people, patients…knocking on windows when nurses close them off…because they couldn’t get heard, and therefore they’d start kicking the window and they’d be injected and taken off to seclusion.’ (Supporter)*

*‘There’s no accountability in these places. The staff are overworked. If somebody’s getting strung out over something it’s just too easy. In fact if they want to actually get rid of them they’ve only got to aggravate him and then they’ve got an excuse to restrain. And that happens, I’ve seen that.’ (Consumer)*

#### Paternalistic attitudes

Paternalism and the importance of achieving compliance with behavioural expectations were also identified as poor practice and inappropriately contributing to the overuse of seclusion and restraint.

*‘To me the restraint is about bringing you into line with a way of thinking about doing what’s best for you…Seclusion and restraint is about compliance.’ (Consumer)*

#### Lack of communication and interaction

Participants noted that most staff did not appear to have the time to talk or interact with inpatients, particularly those who are distressed. Some participants thought some nursing staff were desensitised and uncaring. Others wondered about a lack of appropriate training, or an inability or unwillingness to use recovery-based techniques and the inappropriateness of some treatment environments, particularly the emergency department (ED):

*‘For those nurses in the ED, I mean they haven’t got a clue many of them, you know, and never mind even the nurses in the mental health facilities.’ (Supporter)*

*‘Emergency [department] is just the worst place for mental health issues.’ (Consumer)*

Supporter participants suggested that they often shared the sense of powerlessness that consumers felt. Poor communication from staff across the admission process, family members being prevented access to their loved ones by mental health services and a lack of follow-up from staff after release all contribute to their identification of poor practice.

One supporter from a culturally and linguistically diverse background described their experience of poor communication from mental health services during a time when her husband was being restrained:

*‘I didn’t know what’s wrong when that happen, and just too much for me, and then next thing I call my sister*-*in*-*law and she came with her husband, and then the doctors and nurses start talking to her, and the whole night they didn’t talk to me, I asked them what’s wrong with my husband, and I think it’s discrimination because I’m from overseas.’ (Supporter)*

This participant thought she knew her husband better than anyone in this situation, but that she was not consulted because English was not her first language and because of her cultural background:

*‘…and then she said he sick like this all his life, he’s crazy, and I said no my husband he’s a nice person, he never ever hurt anyone, and then just the way they treat me is horrible, and I think because I think that they didn’t bother consult with me because oh she’s just from overseas.’ (Supporter)*

#### Lack of alternative strategies

Many participants pointed to the use of seclusion and restraint as a first rather than last resort in responding to individuals undergoing a mental health crisis. The lack of de-escalation strategies being used from the initial point of crisis was linked by some participants to the use of restraint:

*‘So what they said is if we’re concerned before they get in the ambulance we’re going to physically restrain them, because that is their number one priority.’ (Supporter)*

Emotional restraint was linked with poor practice. Participants were concerned that withholding privileges can create escalation of tension and agitation, justifying the management of risk through seclusion and restraint:

*‘The only time I’ve ever seen nurses engaged in any kind of de*-*escalation tactics other than seclusion and restraint, is when they’re giving the patient the alternative that they take their sedative willingly or they’re held down, that’s it.’ (Supporter)*

Participants were also invited to talk about why they thought poor practice existed. The following section analyses some of the major themes in this regard.

### Perspectives on what contributes to poor practice

There was a confluence of factors identified by participants as contributing to poor practice, with the main factors being organisational culture, the physical environment, under-resourced mental health services, and fear and ‘stigma’.

#### Organisational culture

The organisational culture and attitudes of mental health services staff were viewed as important contributing factors to poor practice:

*‘Somehow there’s a sanction given to people to be horrible to other people because of the group that they’re a member of. And I think that’s something, if I don’t say another thing, that’s all I want to say.’ (Supporter)*

The acceptance of seclusion and restraint as a first rather than a last resort in responding to people in crisis as well as a lack of training in de-escalation techniques or alternative strategies were all viewed as organisational factors that contributed to poor practice.

#### The physical environment

Some participants pointed to the ‘fishbowl’ ward design in inpatient units as a barrier which not only separates nursing staff from inpatients physically, but also appeared to reinforce separation on an interpersonal level. Others pointed to the lack of a quiet, private space that offered an alternative to a seclusion room:

*‘My son had actually often, when he was admitted, asked to use seclusion as a way of getting away from people and getting some peace.’ (Supporter)*

#### Under-resourced mental health services

Participants pointed to a causal link between mental health services being under-resourced and poor practice. The staff who were on duty were seen as too busy and stressed to attend to consumers’ needs. For example:

*‘You’ll go talk to them and they seem run off their feet and angry and stuff, and they take it, then their mood affects everyone else, because they’re usually carrying on. Sometimes they’re just as bad as people on the ward.’ (Consumer)*

#### Fear and stigma

Fear was seen as a common contributor to the use of seclusion and restraint:

*‘Staff are frightened, police officers are probably frightened too, like people don’t necessarily have those connections, like staff in hospitals don’t always have connections with people that are like deep enough to, or like genuine enough to talk to people when they’re in really bad distress, and I think it’s not necessarily that the staff are really bad, it’s just that there’s not the money for them to spend the time that they would need to spend…I think there’s all that stuff, there’s a culture of fear in Australia like fear of difference, I think that adds to it.’ (Consumer)*

There was also a perception that ‘stigma’ associated with mental health and substance use problems could lead to poor practice. One participant thought that individuals who are drug affected may be seen as undeserving of compassionate attention and therefore unfairly subject to more seclusion and restraint:

*‘I used to always want to be stoned and I think well that’s beyond panic attacks, that’s like I want to be in a coma, that’s how stressed out I am, I want to be like partially conscious…that’s not like something that we should just reject these people, and say…they’ve got these drug problems…they brought it on themselves…that’s the feeling I get when people talk about dual diagnosis.’ (Consumer)*

## Discussion

The themes presented in this paper are based on the questions used in the focus groups to guide discussion and the themes that emerged in the data analysis, which used a general inductive approach [36]. The responses suggest that many participants attended the focus groups in order to express their concerns about poor practice in mental health settings as well as raise concerns about the use of restraints in emergency departments and by the police.

The traumatic impact of seclusion and restraint represents one of the major themes that were apparent across the focus group discussions. Participants identified seclusion and restraint as nontherapeutic, anti-recovery and an abuse of human rights. The traumatic effects of these practices are long-standing and not limited to an acute or inpatient setting. Participants also recognised specific challenges for Indigenous and culturally and linguistically diverse populations. The findings indicate the need for further, specific investigation into the use of seclusion and restraint involving minority and marginalised groups.

Participants gave a number of examples of poor practice, including the use of excessive force, lack of empathy/paternalistic attitudes, lack of communication and interaction and a lack of alternative strategies to the use of seclusion and restraint. There was a confluence of contributing factors to poor practice identified revolving around organisational culture, the physical environment, under-resourced mental health services and fear and ‘stigma’. The latter term was used by participants rather than ‘prejudice’ or ‘discrimination’, but stigma in common parlance could be interpreted as meaning unwarranted negative attitudes as well as the societal codification of such attitudes, rather than locating the problem within the individual as Chamberlin has defined this term [19].

These findings suggest that groups of consumers and their supporters across Australia share similar concerns about the harm caused by the use of seclusion and restraint. The harms identified were viewed as being caused by the intrinsic effects of excessive force, isolation and the breaching of human rights, particularly in relation to the loss of dignity. Such harms were viewed as longstanding for consumers and for supporters and usually (re)traumatising. Participants also raised concerns about the lack communication and interaction in mental health services alongside practices of ‘othering’, paired with stigma and fear. Only one participant expressed the view that seclusion and restraint was a ‘necessary evil’.

These findings indicate that consumers and supporters view ‘poor practice’ as indicative of a system which expects and condones mental health practioners to use seclusion and restraint to manage behaviour, despite consumers experiencing such practices in an overwhelmingly negative way. This suggests that it is institutional cultures and norms that require addressing [15].

The focus groups were deliberately small and based on participants opting into participate. This means that the generalisability of the findings is limited. There were both ethical and financial constraints on the project that limited the potential for more targeted purposive sampling. However, a broad range of participants did attend, the discussions were lengthy and fruitful and a safe environment for the discussion of such potentially sensitive issues was achieved. This was particularly supported by having a co-facilitator who shared the participants’ lived experience.

## Conclusion

As the literature outlined above suggests, the concerns raised by focus group participants in this study are longstanding and cross national boundaries. While there may be support for the need for restraint and seclusion in very limited circumstances, in the main, the participants viewed these practices as unnecessarily overused. While the use of seclusion and restraint may meet an immediate need to control and contain, this also creates and exacerbates problems for consumers, supporters, staff and the broader system of care.

This paper confirms that the lived experience of consumers and supporters can make an important contribution to deepening the understanding of what is happening in mental health practice and what needs to change and why. The shift to taking a recovery oriented approach to practice has raised the imperative to address these ongoing concerns. Otherwise, the reality will continue not to match the rhetoric.

## Abbreviations

ED: emergency department
CALD: culturally and linguistically diverse
