# Erratum to: Consumers and their supporters’ perspectives on poor practice and the use of seclusion and restraint in mental health settings: results from Australian focus groups

**DOI:** 10.1186/s13033-016-0039-9

**Published:** 2016-02-17

**Authors:** Lisa M. Brophy, Catherine E. Roper, Bridget E. Hamilton, Juan José Tellez, Bernadette M. McSherry

**Affiliations:** Centre for Mental Health, Melbourne School of Population and Global Health, University of Melbourne, 4/207 Bouverie Street, Carlton, VIC 3010 Australia; Mind Australia, 86-92 Mount Street, Heidelberg, VIC 3084 Australia; Consumer Academic, Centre for Psychiatric Nursing, School of Health Sciences, University of Melbourne, Level 6 Alan Gilbert Building, 161 Barry Street, Carlton, VIC 3053 Australia; Department of Nursing, School of Health Sciences, University of Melbourne, Level 6 Alan Gilbert Building, 161 Barry Street, Carlton, VIC 3053 Australia; St Vincent’s Mental Health, 41 Victoria Parade, Fitzroy, VIC 3065 Australia; Melbourne Social Equity Institute, University of Melbourne, 201 Grattan Street, Carlton, VIC 3053 Australia; Melbourne Law School, University Square, 185 Pelham Street, Carlton, VIC 3035 Australia; Faculty of Law, Monash University, 15 Ancora Imparo Way, Wellington Road, Clayton, VIC 3800 Australia

## Erratum to: Int J Ment Health Syst (2016) 10:6 DOI 10.1186/s13033-016-0038-x

Due to a publisher error, the original version of this article [1] was regretfully published with an incorrect title. The title should read: Consumers and their supporters’ perspectives on poor practice and the use of seclusion and restraint in mental health settings: results from Australian focus groups. The original article has also been updated to present the correct title.
